# Targeted Deletion of Loxl3 by Col2a1-Cre Leads to Progressive Hearing Loss

**DOI:** 10.3389/fcell.2021.683495

**Published:** 2021-06-04

**Authors:** Ziyi Liu, Xinfeng Bai, Peifeng Wan, Fan Mo, Ge Chen, Jian Zhang, Jiangang Gao

**Affiliations:** School of Life Science and Key Laboratory of the Ministry of Education for Experimental Teratology, Shandong University, Jinan, China

**Keywords:** Loxl3, hearing loss, spiral ligament, extracellular matrix, mouse model

## Abstract

Collagens are major constituents of the extracellular matrix (ECM) that play an essential role in the structure of the inner ear and provide elasticity and rigidity when the signals of sound are received and transformed into electrical signals. LOXL3 is a member of the lysyl oxidase (LOX) family that are copper-dependent amine oxidases, generating covalent cross-links to stabilize polymeric elastin and collagen fibers in the ECM. Biallelic missense variant of LOXL3 was found in Stickler syndrome with mild conductive hearing loss. However, available information regarding the specific roles of LOXL3 in auditory function is limited. In this study, we showed that the Col2a1-Cre-mediated ablation of Loxl3 in the inner ear can cause progressive hearing loss, degeneration of hair cells and secondary degeneration of spiral ganglion neurons. The abnormal distribution of type II collagen in the spiral ligament and increased inflammatory responses were also found in *Col2a1–Loxl3^–/–^* mice. Amino oxidase activity exerts an effect on collagen; thus, Loxl3 deficiency was expected to result in the instability of collagen in the spiral ligament and the basilar membrane, which may interfere with the mechanical properties of the organ of Corti and induce the inflammatory responses that are responsible for the hearing loss. Overall, our findings suggest that Loxl3 may play an essential role in maintaining hearing function.

## Introduction

Collagens are major constituents of the extracellular matrix (ECM) and play an essential role in the structure of the inner ear, providing elasticity, and rigidity when the signals of sound are received and transformed into electrical signals. Several types of collagen have been reported in the inner ear, including types I, II, III, IV, V, IX, and XI (Cosgrove et al., 1996; Tsuprun and Santi, 1997). Among these, type II collagen was expressed in the spiral limbus, tectorial membrane (TM), and basilar membrane and was the most abundant component of the spiral ligament (Thalmann, 1993; Dreiling et al., 2002; Husar-Memmer et al., 2013). Furthermore, the TM is composed mostly of the parallel arrangement of type II collagen bundles and associated with other fibrillar and nonfibrillar types of collagen, such as Collagen V, IX, and XI (Richardson et al., 1987; Legan et al., 1997; Gavara et al., 2011; Andrade et al., 2016). Both type V collagen and type IV collagen were identified in the strial capillary basement membrane (Cosgrove et al., 1996; Meyer zum Gottesberge and Felix, 2005). More and more mutations of different types of collagen have been identified in both syndromic and non-syndromic hearing loss, such as Stickler syndrome (Vikkula et al., 1995; Richards et al., 1996; Williams et al., 1996), Spondyloepiphyseal dysplasia congenita (Mark et al., 2011; Veeravagu et al., 2013; Xu et al., 2014), Marshall syndrome (Griffith et al., 2000), and Alport syndrome (Mochizuki et al., 1994).

Apart from mutations in genes encoding collagen, a homozygous missense variant in LOXL3 (c.2027G>A, p.Cys676Tyr) was found in two siblings with an autosomal recessive Stickler syndrome, one of the siblings exhibited mild conductive hearing loss (Alzahrani et al., 2015). Stickler syndrome is mostly an autosomal dominant human collagenophathy caused by monoallelic mutations in *COL2A1*, *COL11A2*, or *COL11A1* (Robin et al., 1993). Stickler syndrome is also inherited in an autosomal recessive manner caused by pathogenic variants in *COL9A1*, *COL9A2*, or *COL9A3.* The symptoms of Stickler syndrome include retinal detachment, auditory dysfunction, hypermobile tympanic membrane, and joint laxity (McAlinden et al., 2008; Acke et al., 2012). LOXL3 is a member of the LOX family that are copper-dependent amine oxidases. All the members can catalyze the oxidative deamination of lysine and hydroxylysine residues, generating covalent cross-links to convert soluble collagen and elastin chains into the insoluble form and stabilize polymeric elastin and collagen fibers in the ECM (Lucero and Kagan, 2006). In the amine oxidase assay, LOXL3 presents amine oxidase activity toward different types of collagen (types I, II, III, IV, VI, VIII, and X). Additionally, the amino oxidase activity of LOXL3 could be suppressed by β-aminopropionitrile (β-APN), with the inhibition of the formation of covalent cross-links *in vivo* (Lee and Kim, 2006; Jeong and Kim, 2017). Further studies were performed using several animal models to understand the importance of LOXL3 in development. A zebrafish model with the lack of Loxl3b presented craniofacial defects (van Boxtel et al., 2011). These results were consistent with our previous finding. Our results showed that Loxl3-deficient mice (*Loxl3*^–/–^) exhibited perinatal lethality and severe craniofacial defects, containing palatal cleft and shortened mandible. Additionally, the Loxl3^–/–^ mice showed abnormalities in the cartilage primordia of the thoracic vertebrae (Zhang et al., 2015). Both craniofacial defects and spinal cord deformities are related to the decrease of mature collagen cross-links. However, thus far, the effect of LOXL3 on auditory function has not been studied.

Considering the amino oxidase activity of LOXL3 on collagen, Loxl3 conditional knockout mice in the inner ear were generated by crossing homozygous floxed-*Loxl3* mice (*Loxl3*^f/f^) (Zhang et al., 2015) with Col2a1-Cre mice that specifically expressed the Cre recombinase under the Col2a1 promoter (Sakai et al., 2001). In Col2a1-Cre transgenic mice, the Cre recombinase was first detected at E9-E9.5 in the otic vesicle and notochord (Sakai et al., 2001). Using the Col2a1–*Loxl3* conditional knockout mice, we explored the roles of the *Loxl3* gene in the maintenance of auditory function.

## Results

### Col2a1-Cre-Mediated Ablation of the *Loxl3* Gene and *Loxl3* Gene Expression in the Inner Ear

**FIGURE 1:**
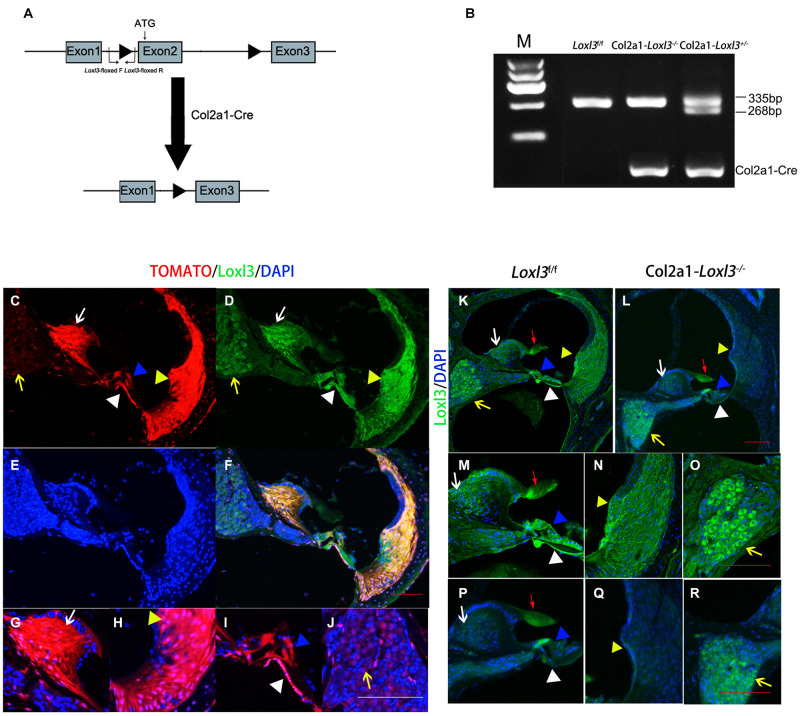
Analysis of Cre recombinase expression in the cochlea of Col2a1-Cre transgenic mice and conditional Loxl3 inactivation in the cochlea of the mice. **(A)** Schematic diagram of the mice containing floxed Loxl3 alleles crossed with Col2a1-Cre transgenic mice. The deletion of Loxl3 exon 2 leads to deleted ATG start codon for protein translation. **(B)** Mouse genotyping using PCR analysis. *Lane1* wild-type (*Loxl3*^f/f^) *Lane2* homozygous (Col2a1–*Loxl3*^–/–^) *Lane3* heterozygous (Col2a1–*Loxl3*^+/–^) mice. **(C–F)** The Cre recombinase expression under the Col2a1 promoter and expression pattern of Loxl3 in mouse cochlea. The expression of Loxl3 and Cre recombinase was overlapped in the spiral limbus (white arrow), spiral ligament (yellow triangle), basilar membrane (white triangle), and hair cells (blue triangle) The Cre recombinase was expressed sparsely in spiral ganglion cells (yellow arrow). Scale bars: 100 μm. **(G–J)** The high magnification of the Cre recombinase expression under the Col2a1 promoter. The expression of Col2a1-Cre recombinase was observed in the most cells of spiral limbus (white arrow), all types of fibrocytes in spiral ligament (yellow triangle), basilar membrane (white triangle), and hair cells (blue triangle). The Cre recombinase was expressed sparsely in spiral ganglion cells (yellow arrow) and not expressed in stria vascularis completely. Scale bars: 100 μm. **(K–L)** The targeted inactivation of Loxl3 in Col2a1–*Loxl3*^–/–^ mice. The targeted inactivation of Loxl3 was observed in the spiral limbus (white arrow), spiral ligament (yellow triangle), basilar membrane (white triangle), and hair cells (blue triangle), except spiral ganglion cells (yellow arrow) and tectorial membrane (red arrow). Scale bars: 100 μm. **(M–O)** The high magnification of the Loxl3 expression. The expression of Loxl3 was observed in the spiral limbus (white arrow), basilar membrane (white triangle), hair cells (blue triangle), tectorial membrane (red arrow), spiral ligament (yellow triangle), and spiral ganglion cells (yellow arrow). **(P–R)** The high magnification of the targeted inactivation of Loxl3 in Col2a1–*Loxl3*^–/–^ mice. The targeted inactivation of Loxl3 was observed in the spiral limbus (white arrow), basilar membrane (white triangle), outer hair cells (blue triangle), and spiral ligament (yellow triangle). Loxl3 was still expressed in tectorial membrane (red arrow), part of inner hair cells and spiral ganglion cells (yellow arrow). Scale bars: 100 μm.

To investigate the role of Loxl3 in inner ear development, Loxl3 conditional knockout mice were generated with the Cre-loxP system. Homozygous mice (*Loxl3*^f/f^) carrying the floxed *Loxl3* allele were crossed with Col2a1-Cre mice (Figure 1A). The genotypes of the pups were identified by PCR (Figure 1B). We analyzed the expression pattern of Cre recombinase under Col2a1 using the *Rosa26-tdTomato* reporter mouse strain (Madisen et al., 2010). At P30, immunofluorescence was performed with sections of the cochlea of the Col2a1-Cre and *Rosa26-tdTomato* doubly transgenic mice (Figures 1C–J). The Cre recombinase under the Col2a1 promoter was expressed in the most of spiral limbus (white arrow), all types of fibrocytes in spiral ligament (yellow triangle), basilar membrane (white triangle), and hair cells (blue triangle). The Cre recombinase was expressed sparsely in spiral ganglion cells (yellow arrow) and not expressed in stria vascularis completely. The result of immunofluorescence staining showed that Loxl3 had been inactivated in the spiral limbus (white arrow), basilar membrane (white triangle), outer hair cells (blue triangle), and spiral ligament (yellow triangle). Loxl3 was still expressed in TM (red arrow), part of inner hair cells and spiral ganglion cells (yellow arrow) (Figures 1K–R).

### Targeted Inactivation of Loxl3 in the Cochlea by Col2a1-Cre Leads to Progressive Hearing Loss and Degeneration of OHCs and IHCs

**FIGURE 2:**
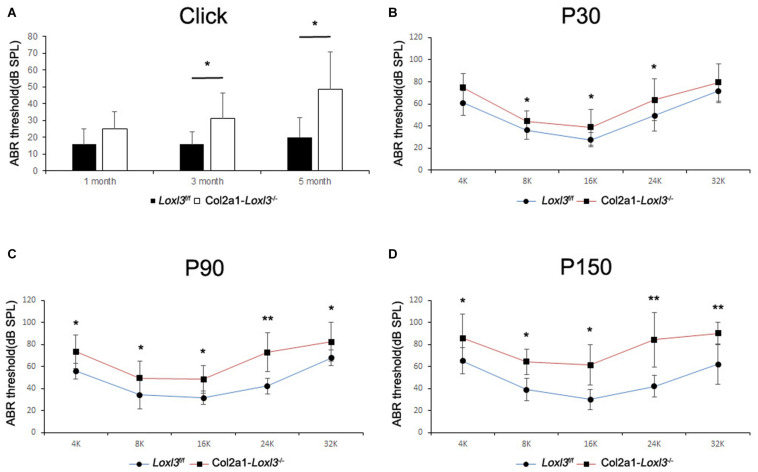
ABR analysis between *Loxl3*^f/f^ and *Col2a1–Loxl3^–/–^* mice. **(A)** ABR thresholds with a broadband click between *Loxl3*^f/f^ and *Col2a1–Loxl3^–/–^* mice (*n* = 10). No obvious difference was observed at P30, and the ABR thresholds were increased significantly at P90 and P150, indicating progressive hearing loss. **(B–D)** ABR thresholds with frequency-specific pure tone stimuli (containing 4, 8, 16, 24, and 32 k) (*n* = 10). The ABR thresholds were increased significantly from P30 to P150. *p < 0.05; **p < 0.01 by two-tailed Student’s *t* test.

To assess the hearing function of *Col2a1–Loxl3*^–^*^/^*^–^ mice, auditory brainstem response (ABR) measurements were performed from P30. In broadband click, adult *Col2a1–Loxl3*^–^*^/^*^–^ mice exhibited no obvious difference in the ABR thresholds at P30. Moreover, the ABR thresholds of the *Col2a1–Loxl3*^–^*^/^*^–^ mice were increased at P90 with a sound pressure level (SPL) of averaged 35 dB, whereas the *Loxl3*^f/f^ mice showed hearing thresholds of 10–20 dB SPL (Figure 2A). And the data showed a statistically significant increase in the ABR thresholds of the *Col2a1–Loxl3*^–^*^/^*^–^ mice. At P150, the *Col2a1–Loxl3*^–^*^/^*^–^ mice exhibited variation of ABR thresholds of 30–70 dB SPL, and the average of ABR thresholds for broadband click was significantly elevated to approximately 50 dB compared to 20 dB in *Loxl3*^f/f^ mice.

The ABR measurements with frequency-specific pure tone stimuli also showed a progressive increase in the ABR thresholds of *Col2a1–Loxl3*^–^*^/^*^–^ mice that were 20–40 dB higher than those in *Loxl3*^f/f^ mice until P150 (Figures 2B–D). The results indicate that the targeted inactivation of Loxl3 under Col2a1-Cre leads to progressive hearing loss.

**FIGURE 3:**
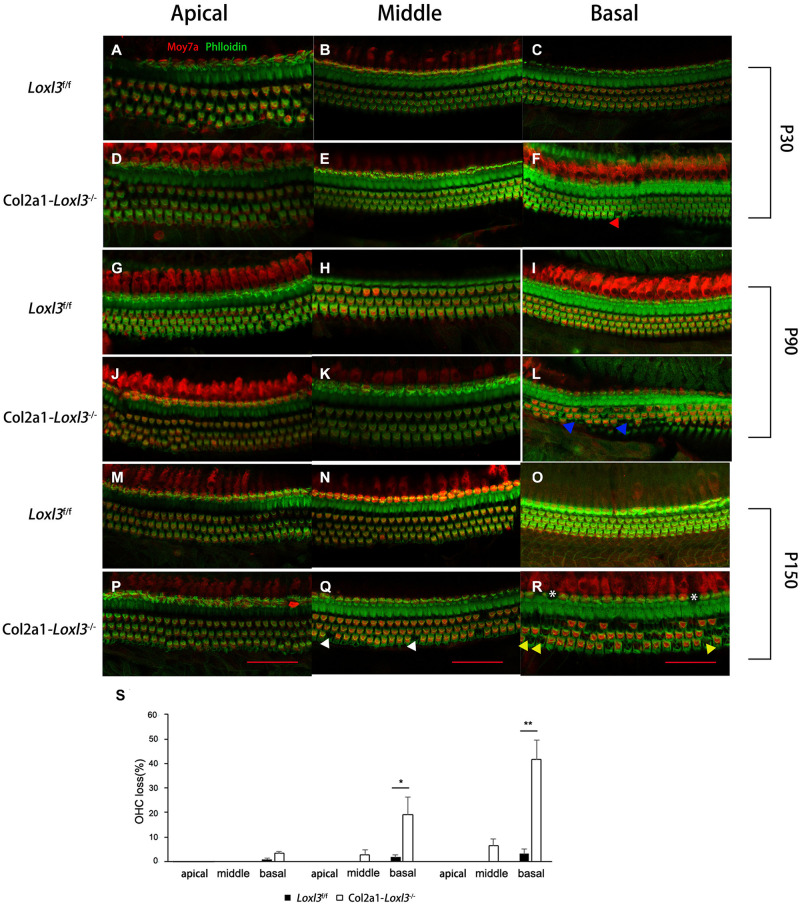
Progressive degeneration of OHCs and IHCs in *Col2a1–Loxl3^–/–^* mice. **(A–F)** Confocal images of cochlea hair cells in *Loxl3*^f/f^ and *Col2a1–Loxl3^–/–^* mice at P30. Spotty loss of OHCs was observed in the cochlea basal turn (red triangle). **(G–L)** Confocal images of cochlea hair cells in *Loxl3*^f/f^ and *Col2a1–Loxl3^–/–^* mice at P90. Approximately 19% OHCs loss was observed in the cochlea basal turn. (blue triangle). **(M–R)** Confocal images of cochlea hair cells in *Loxl3*^f/f^ and *Col2a1–Loxl3^–/–^* mice at P150. Significant OHCs degeneration was observed in the cochlea basal turn (yellow triangle), with spotty loss of OHCs in the cochlea middle turn (white triangle). And a little loss of IHCs was also observed in the cochlea basal turn (white asterisk) Scale bars: 100 μm. OHCs, outer hair cells; IHCs, inner hair cells. **(S)** Quantifications of OHC loss at specific locations of the cochlea in *Loxl3*^f/f^ and *Col2a1–Loxl3^–/–^* mice (*n* = 10). *p < 0.05; **p < 0.01 by two-tailed Student’s *t* test.

To examine the morphology of hair cells in *Col2a1–Loxl3*^–^*^/^*^–^ mice, immunofluorescence assay of cochlear whole mounts was performed and was labeled with phalloidin (green) for stereociliary bundles and Moy7a (red) for hair cell body. Consistent with the results of ABR measurement, few loss of outer hair cells (OHCs) were found at P30 (Figures 3A–F) and approximately 19% OHC loss was observed in the cochlea basal turn of *Col2a1–Loxl3*^–^*^/^*^–^ mice at P90 (Figures 3G–L). Until P150, significant OHC degeneration was observed in the cochlea basal turn (40%) with spotty loss of OHCs in the cochlea middle turn (6%) of *Col2a1–Loxl3*^–^*^/^*^–^ mice (Figures 3M–R). The data showed statistically significant differences (Figure 3S). And a small amount of IHCs was also absent in the cochlea basal turn (white asterisk).

### Loss of Loxl3 Leads to Impaired Stereocilia of the OHCs and IHCs in the Cochlea

**FIGURE 4:**
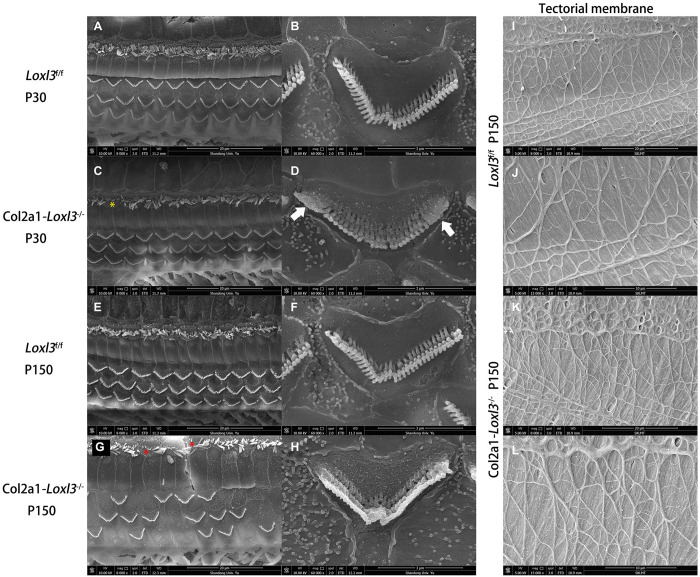
The impaired stereocilia of the outer hair cells and inner hair cells and normal tectorial membrane in *Col2a1–Loxl3^–/–^* mice. **(A–D)** Scanning electron micrograph of hair cell stereocilia in the basal turn of the *Loxl3*^f/f^ and *Col2a1–Loxl3^–/–^* cochlea at P30. The magnification of the outer hair cells (OHC) in *Col2a1–Loxl3^–/–^* mice showed partial stereocilia fusion (white arrow). And the stereocilia of inner hair cells in *Col2a1–Loxl3^–/–^* mice showed spotty loss (yellow asterisk). **(E–H)** Scanning electron micrograph of hair cell stereocilia in the basal turn of the *Loxl3*^f/f^ and *Col2a1–Loxl3^–/–^* cochlea at P150. The stereocilia of the outer hair cells (OHCs) in *Col2a1–Loxl3^–/–^* mice showed serious impairment and complete stereocilia fusion, along with loss of OHCs. And the stereocilia of inner hair cells in *Col2a1–Loxl3^–/–^* mice showed spotty loss (red asterisk). **(I–L)** Scanning electron micrograph of tectorial membrane in the basal turn of the *Loxl3*^f/f^ and *Col2a1–Loxl3^–/–^* cochlea at P150. The magnification of the tectorial membrane ^TM^ in *Col2a1–Loxl3^–/–^* mice showed normal.

To examine the stereocilia of the IHCs and OHCs, scanning electron micrography was performed from the apical turn to the basal turn of the cochlea. At P30, the shape and arrangement of the stereocilia were normal in the *Col2a1–Loxl3*^–^*^/^*^–^ mice (Figures 4A,C), consistent with the results of ABR measurement and immunofluorescence. However, the high magnification of the OHCs in the basal turn of the *Col2a1–Loxl3*^–^*^/^*^–^ mice cochlea showed partly stereocilia fusion (Figures 4B,D). Until P150, the stereocilia of the OHCs in the basal turn of the *Col2a1–Loxl3*^–^*^/^*^–^ mice cochlea showed serious impairment and complete fusion, (Figures 4F,H) along with loss of OHCs (Figures 4E,G). The stereocilia of IHCs in *Col2a1–Loxl3*^–^*^/^*^–^ mice also showed spotty loss (red asterisk). The high magnification of the TM in scanning electron micrograph of *Col2a1–Loxl3*^–^*^/^*^–^ mice showed normal morphology at P150 (Figures 4I–L).

### *Col2a1–Loxl3*^–^*^/^*^–^ Mice Showed Progressive Degeneration of SGNs

**FIGURE 5:**
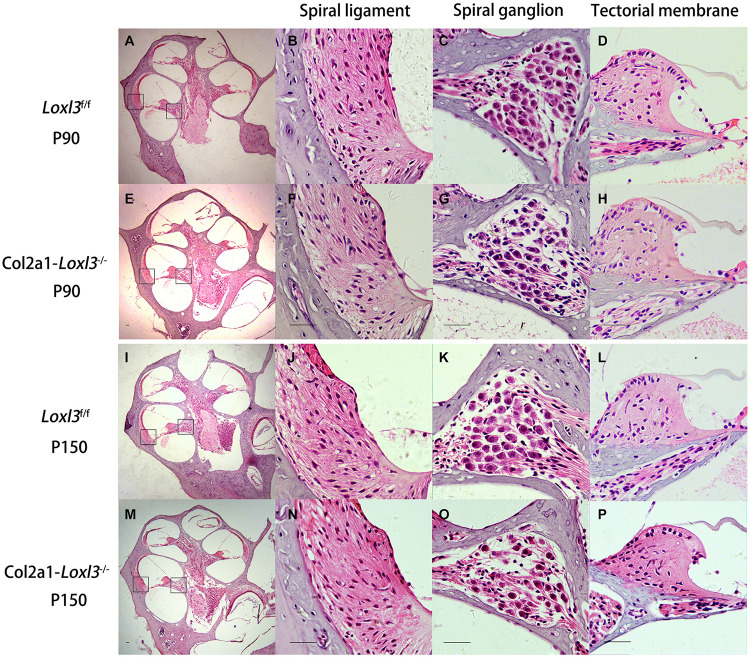
Normal cochlear morphology and progressive degeneration of spiral ganglion neurons (SGNs) in *Col2a1–Loxl3^–/–^* mice. **(A–H)** Hematoxylin and eosin (H&E) staining of sections of *Loxl3*^f/f^ and *Col2a1–Loxl3^–/–^* mice at P90. No significant abnormality was observed in the spiral ligament, where Loxl3 was conditionally inactivated. **(I–P)** Hematoxylin and eosin staining of sections of *Loxl3*^f/f^ and *Col2a1–Loxl3^–/–^* mice at P150. The progressive degeneration of the SGNs appeared in the cochlea basal turn of *Col2a1–Loxl3^–/–^* mice at P150. Scale bars: 200 μm.

**FIGURE 6:**
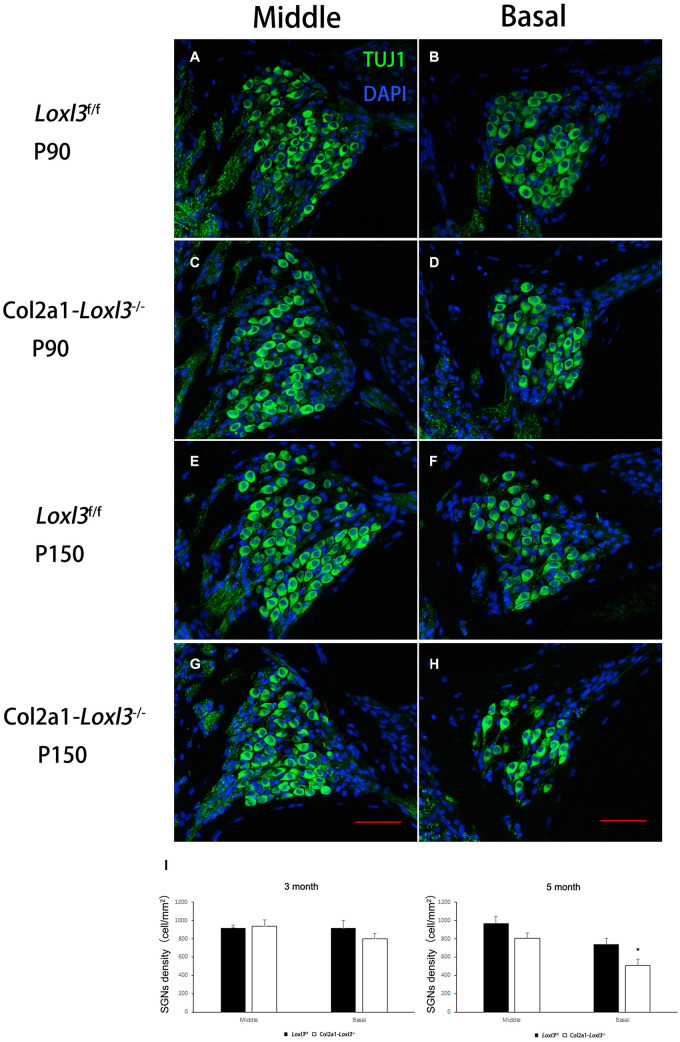
Images of SGNs in *Loxl3*^f/f^ and *Col2a1–Loxl3^–/–^* mice. **(A–D)** Representative image of Tuj1 (green) immunofluorescence in the spiral ganglion cells of *Loxl3*^f/f^ and *Col2a1–Loxl3^–/–^* mice at P90. Tuj1 is a marker of types I and II neurons; no significant difference was observed between the *Col2a1–Loxl3^–/–^* and *Loxl3*^f/f^ mice. **(E–H)** Representative image of Tuj1 (green) immunofluorescence in the spiral ganglion cells of *Loxl3*^f/f^ and *Col2a1–Loxl3^–/–^* mice at P150. Scale bars: 100 μm. **(I)** Counting of the SGNs in the serial sections showed a significant decrease in the density of the SGNs in *Col2a1–Loxl3^–/–^* mice at P150 (*n* = 5). *p < 0.05 by two-tailed Student’s *t* test.

The morphology of the cochlea in the inner ear was investigated by hematoxylin and eosin (H&E) staining. There was no apparent abnormal cochlear morphology in *Col2a1–Loxl3*^–^*^/^*^–^ mice at P90 (Figures 5A–H), even in the spiral limbus and spiral ligament, where the Loxl3 was inactivated at P150 (Figures 5I–P). The TM of *Col2a1–Loxl3*^–^*^/^*^–^ mice was also normal at P90 and P150 (Figures 5H,P). However, the spiral ganglion neurons (SGNs) of *Col2a1–Loxl3*^–^*^/^*^–^ mice appeared degenerated at P150; this may be due to the loss of OHCs (Figure 5O). The immunofluorescence microscopy of Tubulin β3 (Tuj1), a marker for both types I and II SGNs, was performed (Figures 6A–H). The SGNs data by the serial sections showed that the density of SGNs decreased significantly in *Col2a1–Loxl3*^–^*^/^*^–^ mice at P150 (Figure 6I).

### *Col2a1–Loxl3*^–^*^/^*^–^ Mice Showed Abnormal Distribution of Type II Collagen in Spiral Ligament and Increased Inflammatory Responses

**FIGURE 7:**
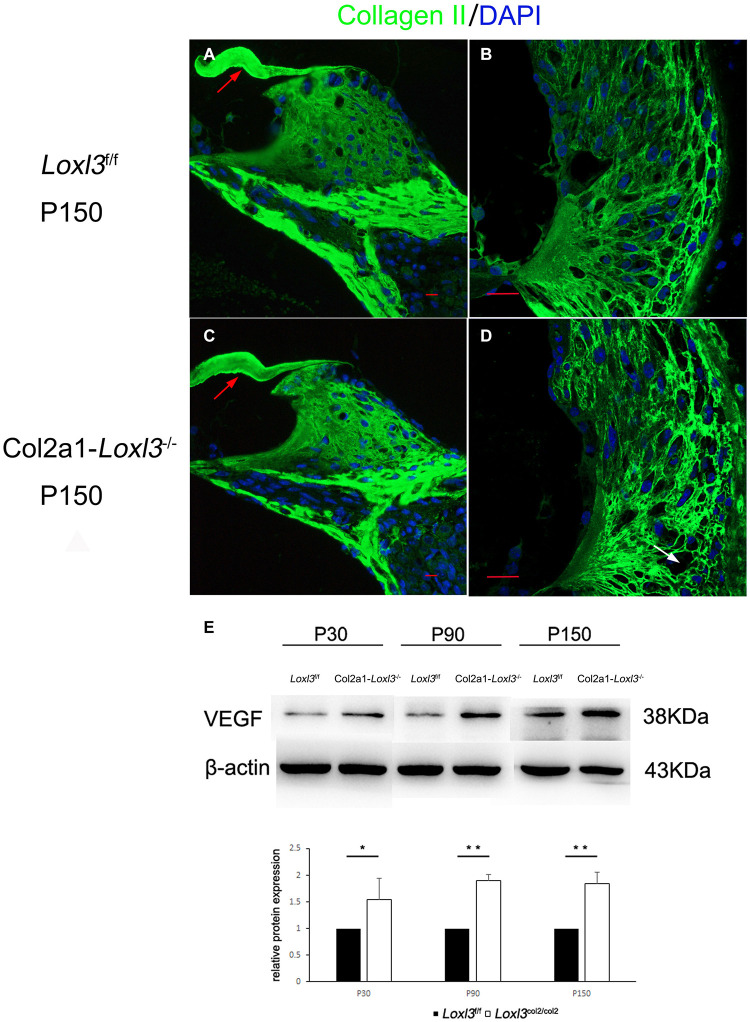
Distribution of Type II collagen in the spiral ligament and the expression of vascular endothelial growth factor (VEGF) in the cochlea of *Loxl3*^f/f^ and *Col2a1–Loxl3^–/–^* mice. (**A**,**C**) Representative image of type II collagen (green) immunofluorescence in the spiral limbus and the tectorial membrane of *Loxl3*^f/f^ and *Col2a1–Loxl3^–/–^* mice at P150. The deposition of type II collagen was normal in the tectorial membrane of *Col2a1–Loxl3^–/–^* mice than in those of *Loxl3*^f/f^ mice (red arrow). (**B**,**D**) Representative image of type II collagen (green) immunofluorescence in the spiral ligament of *Loxl3*^f/f^ and *Col2a1–Loxl3^–/–^*mice at P150. The deposition of type II collagen was slightly abnormal in the spiral ligament of *Col2a1–Loxl3^–/–^* mice than in those of *Loxl3*^f/f^ mice (white arrow). Scale bars: 20 μm. **(E)** Western blot analysis of VEGF expression in the cochlea of *Col2a1–Loxl3^–/–^* and *Loxl3*^f/f^ mice at P30–P150. The results of western blot analysis showed that the VEGF expression was significantly increased in the cochlea of *Col2a1–Loxl3^–/–^* mice than in those of *Loxl3*^f/f^ mice at P30–P150 (*n* = 5; *p < 0.05; **p < 0.01).

The primary function of Loxl3 is to catalyze the covalent cross-links of collagen, and type II collagen is the most abundant in the whole cochlea (Husar-Memmer et al., 2013). Thus, the distribution of type II collagen was examined in the inner ear. Immunofluorescence staining revealed that the distribution of type II collagen was normal in the TM and spiral limbus of *Col2a1–Loxl3*^–^*^/^*^–^ mice as compared with that in *Loxl3*^f/f^ mice (Figures 7A,C). However, the type II collagen fibrils were slightly abnormal in the spiral ligament of *Col2a1–Loxl3*^–^*^/^*^–^ mice than in those of *Loxl3*^f/f^ mice. Sparse and curved type II collagen fibrils were observed (Figures 7B,D). Type II collagen plays an essential role in the spiral ligament; thus, the function of the spiral ligament fibrocytes may be affected, which are proposed to be associated with the mediation of inflammatory responses to trauma. To detect the inflammatory responses in the cochlea of *Col2a1–Loxl3*^–^*^/^*^–^ mice and *Loxl3*^f/f^ mice, vascular endothelial growth factor (VEGF), a marker of the inflammatory responses, was analyzed using western blot. The results of western blot analysis showed that the VEGF expression in the cochlea of *Col2a1–Loxl3*^–^*^/^*^–^ mice was significantly higher than in those of *Loxl3*^f/f^ mice at P30–P150, implying the inflammatory responses in the cochlea of *Col2a1–Loxl3*^–^*^/^*^–^ mice (Figure 7E).

## Discussion

As demonstrated by the perinatal lethality of the Loxl3-null mice (Zhang et al., 2015), Lox3 plays an critical role in embryonic development. Although a variety novel functions of Loxl3 have been described, the most important biological function of LOXL3 is related to the amino oxidase activity for the crosslinking of collagen.

In this study, we showed that Col2a1-Cre-mediated *Loxl3* ablation in the inner ear can cause progressive hearing loss and degeneration of OHCs and IHCs. At P150, the average of ABR thresholds for broadband click in Col2a1*–Loxl3*^–^*^/^*^–^ mice was significantly elevated to approximately 50 dB compared with 20 dB in *Loxl3*^f/f^ mice. Moreover, no apparent morphologic abnormality was found at the light microscopic level in whole mounts and cross-sections of inner ears. However, the SGNs in *Col2a1–Loxl3*^–^*^/^*^–^ mice appeared degenerated at P150; this may be caused by the loss of OHCs. An irreversible damage to cochlear hair cells is often followed by secondary degeneration of SGNs (Dodson and Mohuiddin, 2000; Xu et al., 2021). The degeneration of SGNs usually starts with the loss of synaptic terminals followed by the disintegration of cell bodies (Pan et al., 2011; Shibata et al., 2011).

In the previous study, we crossed the *Loxl3*^f/f^ mice with Atoh1-Cre mice that primarily express the Cre recombinase in hair cells, and the mice with deletion of Loxl3 mediated by Atoh1 promotor did not show hearing loss and degeneration of OHCs (data not shown), suggesting that the precise ablation of Loxl3 in hair cells was not able to affect the function of OHCs. The results of this study showed that the expression of Cre recombinase in Col2a1-Cre transgenic mice overlapped with the expression pattern of Loxl3, including the spiral limbus, basilar membrane, and spiral ligament; hence, the primary function of Loxl3 in the inner ear may be to catalyze the cross-links of collagen. Moreover, the deficiency of Loxl3 was expected to result in collagen instability that leads to the semblable phenotypes in *Col2a1–Loxl3*^–^*^/^*^–^ mice and several mouse models with mutations of collagen, such as progressive hearing loss in type IX collagen knockout mice (Suzuki et al., 2005).

Among the several types of collagen expressed in the inner ear, type II collagen is the most abundant throughout the cochlea; it is encoded by Col2a1 and comprises three identical alpha-1(II) chains (Husar-Memmer et al., 2013). The results of Type II collagen immunofluorescence showed that the deposition of type II collagen was basically in the TM, spiral limbus and spiral ligament. The type II collagen fibrils were regular in the TM and spiral limbus, consistent with the results of H&E staining. However, the type II collagen fibrils were sparse and curved in the spiral ligament of *Col2a1–Loxl3*^–^*^/^*^–^ mice compared with those in *Loxl3*^f/f^ mice. Type II collagen is produced by spiral ligament fibrocytes, and the abnormal staining could be a sign of pathology (Tsuprun and Santi, 1999; Buckiova et al., 2006). Type II collagen is also the main component of the spiral ligament where collagen fibrils provide stability and strength to the ECM and maintain the integrity of the ion transport systems (Spicer and Schulte, 1991). As shown by the results, the expression of Loxl3 was basically deleted in the spiral ligament; which might affect the crosslink of collagen and led to abnormal type II collagen fibrils. Then the function of spiral ligament was affected.

Western blot analysis showed that the VEGF expression was significantly increased in the cochlea of *Col2a1–Loxl3*^–^*^/^*^–^ mice than in those of *Loxl3*^f/f^ mice at P30–P150. It had been reported that VEGF expression was upregulated at the onset of cochlear noise-induced damage. The VEGF upregulation was observed in the stria vascularis, spiral ligament and spiral ganglion and the expression of VEGF-receptors was normal (Picciotti et al., 2006). Therefore, these pathological phenotypes of *Col2a1–Loxl3*^–^*^/^*^–^ mice were similar to the cochlear damage induced by noise or ototoxic antibiotics (Jiang et al., 2017; Sha and Schacht, 2017). Moreover, the death of hair cells and SGNs induced by noise or ototoxic antibiotics was mostly associated with macrophage recruitment and cochlear inflammation (Kaur et al., 2018; He et al., 2020). In noise-induced hearing loss, the damage to fibrocytes of the spiral ligament is likely to lead to changes in cytokines or chemokines such as TNF-a, IL-1b, IL-6, and Icam-1 (Fujioka et al., 2006, 2014; Tornabene et al., 2006). The cochlear spiral ligament is a connective tissue that is suggested to play an essential role in the pathophysiology of different etiologies of hearing loss. The spiral ligament is composed of several specialized fibrocytes that are proposed to play different roles in fluid homeostasis, the mediation of inflammatory responses to trauma, and the fine tuning of cochlear mechanics (Hequembourg and Liberman, 2001; Wangemann, 2006; Ohlemiller, 2009). VEGF could induce vascular neogenesis and was produced by spiral ligament fibrocytes stimulated by IL-1L or TNF-K. These inflammatory response mediators together might induce inflammatory cell movement that would prolong the inflammatory response and impair hair cells (Yoshida et al., 1999). Thus, the abnormal type II collagen fibrils caused by inactivated Loxl3 may affect the function of spiral ligament and lead to inflammatory response in inner ear of Col2a1*–Loxl3*^–^*^/^*^–^ mice, resulting in the loss of hair cells and secondary degeneration of SGNs. Then the Col2a1*–Loxl3*^–^*^/^*^–^ mice showed progressive hearing loss.

Type II collagen has also been detected in the basilar membrane, overlapping with the deletion of Loxl3 in *Col2a1–Loxl3*^–^*^/^*^–^ mice. Both sensory hair cells and supporting cells are localized on the basilar membrane; Deiters’ and pillar cells are essential for constituting a cochlear amplifier, providing necessary structural support for the hair cells (Cosgrove et al., 1996; Meyer zum Gottesberge and Felix, 2005). It has been discovered that the deficiency of one collagen-binding receptor (DDR1) in mice leads to both the abnormal morphology of the supporting cells and the alteration of OHCs. It is noteworthy that the DDR1-null mice also showed ABR thresholds shift at P60, explaining that the variation in intracellular electron density of Deiters’ cells led to the reduced anchorage to the basilar membrane and impairment in OHCs (Meyer zum Gottesberge et al., 2008). In *Col2a1–Loxl3*^–^*^/^*^–^ mice, the structural abnormality of the basilar membrane and Deiters’ cells lead to the degeneration of OHCs, and the ultrastructural features of the basilar membrane are expected to be observed in further investigation.

In conclusion, we have demonstrated that Loxl3 plays an essential role in the maintenance of the inner ear function; the results may provide more explanation for the pathology of Stickler syndrome caused by homozygous mutations in LOXL3.

## Materials and Methods

### Animals

*Rosa26-tdTomato* (No. 007914) and Col2a1-Cre (No. 003554) mice were obtained from the Jackson Laboratory. Mice homozygous for floxed Loxl3 exon2 were crossed with Col2a1-Cre mice. The following primers were used for mouse genotyping: *Loxl3*-floxed F (5′-CCCTTCCTGTCACA TCCTGT-3′) and *Loxl3*-floxed R (5′-AACAGGCACAGCCCT AGAGA-3′) for the floxed Loxl3 allele and Cre-F (5′-GCATC GACCGGTAATGCAGGC-3′) and Cre-R (5′-AGGGTCCAG CCCGAGCTACTT-3′) for specific Col2a1-Cre.

### Auditory Brainstem Response Measurement

Auditory brainstem response was performed in a soundproof room. The mice were anesthetized with pentobarbital sodium (50 mg/kg body weight) via intraperitoneal injection. Three electrodes were inserted subcutaneously in the mice through the cranial vertex, the external ear, and the back near the tail. Brand click and tone burst stimuli of 4, 8, 16, 24, and 32 kHz were generated, and signals of responses were recorded using a Tucker Davis Technologies (TDT, United States) workstation running SigGen32 software (TDT, United States). The ABR thresholds were determined using the lowest sound intensity at which the first wave could be elicited clearly.

### Immunostaining Analysis

The cochlea was fixed in 4% formaldehyde at 4°C overnight and decalcified in 10% EDTA at room temperature for at least 24 h. For sectioning, the cochleae were dehydrated with 15% sucrose for 2 h and later in 30% sucrose overnight at 4°C. Samples were embedded in Tissue-Tek OCT compound and frozen at −20°C and then sectioned into 9-μm-thick slices. For whole-mount immunostaining, the sensory epithelium in the cochlea was isolated and divided into several parts of apical, middle, and basal turns. Then, the sections or whole cochlea samples were permeabilized with 0.5% Triton X-100 in PBS at room temperature for 15 min, washed in PBS thrice, and then blocked in 10% goat serum in PBS at 374°C for 30 min. The samples were incubated with a primary antibody at 4°C overnight. After washing with PBS, further incubation with a secondary antibody (goat anti-rabbit Alexa-488 or Alexa-568, 1:500; Invitrogen) diluted in PBS at 37°C for 1 h was performed, followed by Alexa Fluor 488-conjugated phalloidin (Sigma-Aldrich, United States) at 37°C for 30 min and DAPI at RT for 5 min. Immunofluorescence images were collected using an LSM 880 confocal microscope (Zeiss). The primary antibodies included rabbit anti-LOXL3 (1:200, American Research Products, United States), rabbit anti-myosin VIIa (1:200, Cell Signaling, United States), and rabbit anti-Tuj1 (1:400, Cell Signaling, United States).

### Scanning Electron Microscopy

The inner ears of the mice were dissected and fixed with 2.5% glutaraldehyde overnight at 4°C and decalcified in 10% EDTA at room temperature for at least 24 h. The sensory epithelium in the cochlea was isolated and post-fixed in 1% osmium tetroxide for 2 h. The samples were dehydrated through a graded ethanol series and critically point dried. The samples were then mounted and sputter-coated with gold. Thereafter, stereociliary bundles and TM were examined using a Hitachi S-4800 Field Emission scanning electron microscope.

### Histology Analysis

The cochlea samples were fixed and decalcified using a similar procedure as described in the immunostaining assay; then, they were dehydrated with an ethanol series ranging from 30 to 100% and embedded in paraffin to then be sectioned at a thickness of 5 μm and stained using H&E.

### Western Blot

*Loxl3*^f/f^ and *Col2a1–Loxl3^–/–^* mice were sacrificed via cervical dislocation, and their cochleae were dissected. Cochleae proteins were incubated in cell lysis buffer (P0013, Beyotime) and extracted using a homogenizer. Western blot analyses were performed as described previously (Hou et al., 2014). The following primary antibodies were used: anti-VEGFA polyclonal antibody (rabbit, 1:400, ABclonal) and anti-β-actin polyclonal antibody (rabbit, 1:3,000, Proteintech). All data are presented as mean ± standard error of the mean, and data analyses were performed using the Image J software. Student’s *t* test was used for single-factor experiments involving two groups. The significance level was set to *p* < 0.05 for all statistical analyses.

### Statistical Analysis

Data were expressed as the mean ± SD and indicated from at least three independent experiments. Statistical analysis of the data was performed using a two-tailed-distribution Student’s *t* test or one-way ANOVA using GraphPad Software. For all tests, a value of *p* < 0.05 was considered to be statistically significant.

## Data Availability Statement

The original contributions presented in the study are included in the article/supplementary material, further inquiries can be directed to the corresponding authors.

## Ethics Statement

The animal study was reviewed and approved by Ethics Committee of Shandong University. All experimental procedures about animals were approved by Ethics Committee of Shandong University. Animal management was performed strictly in accordance with the standards of the Animal Ethics Committee of Shandong University (Permit Number: ECAESDUSM 20123004).

## Author Contributions

ZL: writing–original draft, investigation, software, and writing–review and editing. XB: writing–original draft, investigation, and software. PW, FM and GC: investigation. JZ: funding acquisition and writing–review and editing. JG: writing–review and editing, funding acquisition, supervision, resources, project, and administration. All authors contributed to the article and approved the submitted version.

## Conflict of Interest

The authors declare that the research was conducted in the absence of any commercial or financial relationships that could be construed as a potential conflict of interest.
